# Survival from alcoholic hepatitis has not improved over time

**DOI:** 10.1371/journal.pone.0192393

**Published:** 2018-02-14

**Authors:** Emily Hughes, Laurence J. Hopkins, Richard Parker

**Affiliations:** 1 Leeds Liver Unit, St James’s University Hospital, Leeds, United Kingdom; 2 University Hospitals Birmingham NHS Foundation Trust, Birmingham, United Kingdom; 3 University of Birmingham, Birmingham, United Kingdom; Copenhagen University Hospital, DENMARK

## Abstract

**Purpose/Background:**

We aimed to describe changes in survival in alcoholic hepatitis (AH) over time by examining published data.

**Methods:**

A systematic literature search of Ovid Embase and PubMed was undertaken using the MESH terms ‘hepatitis, alcoholic’ to identify randomised controlled trials (RCT) and observational studies (OS) in alcoholic hepatitis. Data were extracted from included studies regarding 28-day, 90-day, 180-day mortality, as well as biochemical and clinical data.

**Results:**

After review of the literature search results, 77 studies published between 1971 and 2016 were analysed, which included data from a total of 8,184 patients. Overall mortality from AH was 26% at 28 days, 29% at 90 days and 44% at 180 days after admission. No changes in mortality over time were observed in univariable analysis at 28 days or 90 days after admission (Pearson correlation r -0.216, p = 0.098, and r 0.121 p = 0.503 respectively). A small but statistically significant increase in mortality was seen in 180-day mortality (r 0.461 p = 0.036). However, after meta-regression to adjust for other factors associated with mortality at each time point, no changes in mortality were seen. Sub-group analysis did not reveal any changes in mortality over time in different study types, or when only biopsy-proven or severe disease were considered.

**Conclusion:**

There has been no improvement in mortality from AH. This is not explained by changes in severity of disease. This emphasises the urgent need for effective treatments for this alcoholic hepatitis.

## Systematic review: Trends in survival from alcoholic hepatitis

Alcohol related liver disease (ArLD) is a common liver disease [1] and the leading cause of liver-related deaths in the UK [2]. ArLD is a spectrum of disease ranging from simple steatosis to cirrhosis [3]. Alcoholic hepatitis (AH) is the most florid manifestation of ArLD, typified by acute jaundice and coagulopathy [4]. In spite of years of research AH is difficult to treat. Many agents have been examined over the years without conspicuous success in the treatment of AH [5–12]. Most recently the largest randomised controlled trial (RCT) in the field of ArLD failed to show a clear benefit of corticosteroid therapy [13]. A small positive benefit on early mortality was found in network meta-analysis of several trials [14], however this was not supported by the most recent direct meta-analysis [15], and any short-term benefits of corticosteroid are not maintained in the longer-term.

In other areas of hepatology for example in acute liver failure [16], hepatocellular carcinoma and viral hepatitis [17], medical not maintained in longer-term de recent reviews of therapy for alcoholic hepatitis, published in 2015 and 2017 respectadvances have caused survival to improve markedly over time. Similarly, decreases in mortality have been observed in common non-hepatic disease such as acute myocardial infarction [18,19] and stroke [20,21]. We used systematic review to examine the mortality of AH in trials and observational studies of AH to evaluate changes over time.

## Methods

A systematic review of studies in alcoholic hepatitis was undertaken, by searching PubMed (1946–2016) EMBASE (1947 to 2016) using the search terms: (((alcoholic hepatitis) NOT non-alcoholic) NOT B0 NOT C, limited to human studies and English language. Furthermore, ‘grey literature’ was examined by looking at reference lists of included studies for relevant manuscripts (snowballing), and articles citing included studies were also reviewed (reverse snowballing). Studies were included if they reported on patients with a clinical or histological diagnosis of alcoholic hepatitis and provided information on mortality at 28 days, 90 days or six months after admission. Only full papers published in English were considered for inclusion. This protocol was not registered.

Data regarding mortality, biochemical severity (bilirubin, prothrombin time or INR, creatinine, white cell count) and participant characteristics (gender and age) were sought. These data were extracted from included papers and entered into a pre-prepared spreadsheet. Two authors (EH and LH) collected data independently and then combined data were checked by all authors to identify inconsistencies. These were resolved by consensus. Missing data were not imputed. The primary outcome considered was mortality from AH. Mortality over time was calculated by considering the year of publication of included studies as a single time point, rather than duration of a study. Overall mortality was noted, and in controlled trials, the mortality in intervention and control groups in each study was also considered. We noted age, alcohol intake, gender and laboratory measurements (bilirubin, creatinine, urea, prothrombin time and white cell count). Where values were reported only for individual groups, overall mean was calculated by the following formula: overall mean = [(n_group 1_/n_total_).(mean_group 1_)]+[(n_group 2_/n_total_).(mean_group 2_)]. When the same cohort or overlapping data collection periods were reported in separate papers, the cohort was included only once and the larger report included. The risk of bias in included studies was assessed using the Cochrane risk of bias tool for randomised studies [22] or the NIH tool for assessing bias in observational studies [23] as appropriate.

Mortality at each time point was calculated as percentages. Correlation of mortality and time was examined in univariable analysis using r^2^ values. Meta-regression analysis using linear regression was used to take into account other factors associated with mortality. Sensitivity analyses of groups of differing types of study (randomised trials vs. observational studies), biopsy-controlled studies and studies incudling patients with severe disease (defined by the discriminant function, DF) were undertaken in the same way. Statistical analysis was performed with Stata version 23 (IBM, New York, USA).

## Results

**Fig 1:**
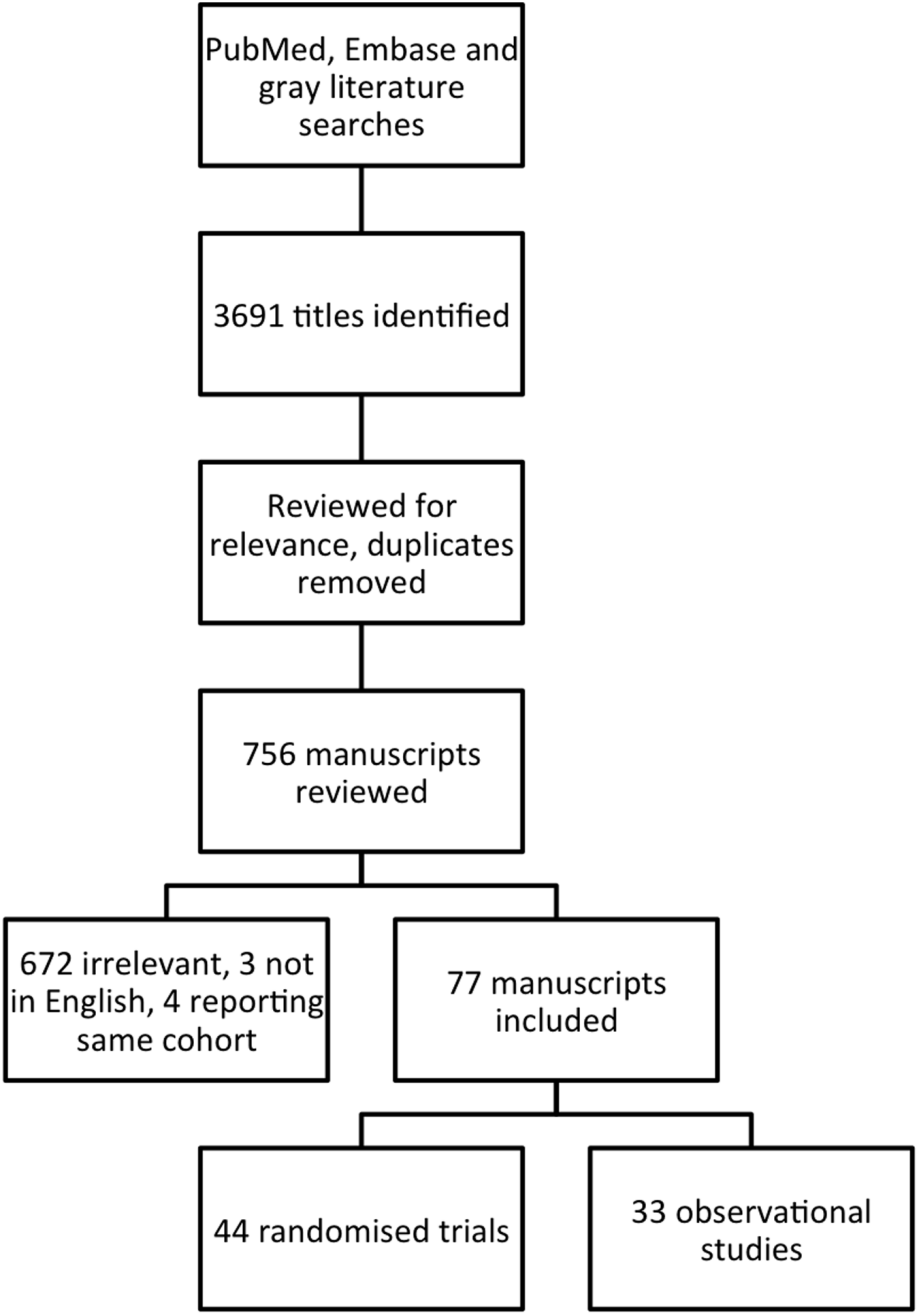
Study flowchart demonstrating literature search process.

The literature search identified 3,691 papers. After exclusion of duplicate search results and irrelevant or ineligible results a final group of 77 papers was included of which 44 were trials of experimental treatments and 33 were observational studies (Fig 1). Of the observational studies, 29 were cohort studies and 4 were case-control studies. These studies described a total of 8,184 participants– 4,100 in RCT and 4,084 in observational studies. The characteristics of each study are summarised in S1–S5 Tables with references to individual studies. The risk of bias was assessed in each study using established methods (S6 and S7 Tables).

Missing data was considerable, particularly regarding mortality beyond 28 days. The overall number of participants contributing to each outcome of interest in summarised in S8 Table. Diagnosis of alcoholic hepatitis was usually based on clinical grounds, particularly in observational studies. Biopsy was more common in trials including patients with AH but not universal. In total 2215 cases were biopsy-proven, representing 33% of all participants.

### Mortality over time

**Fig 2:**
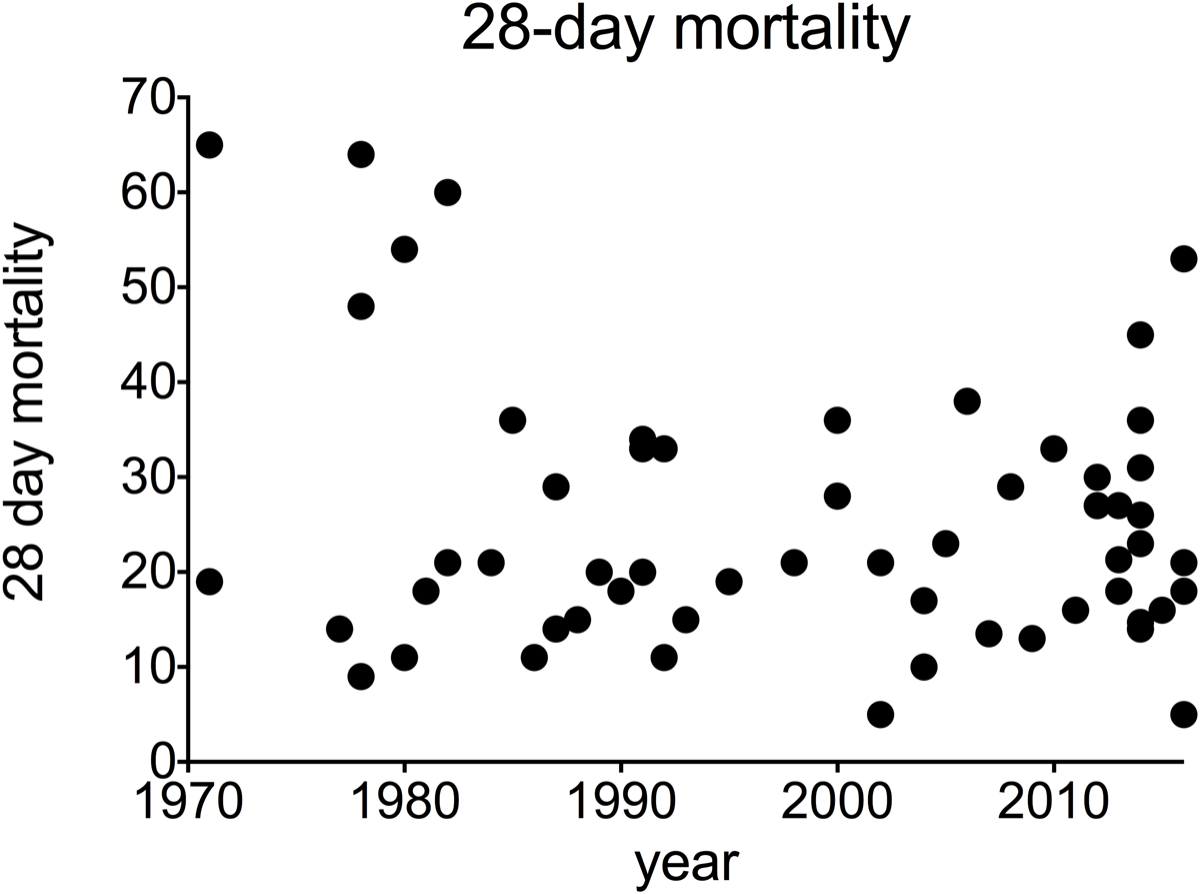
28-day mortality in alcoholic hepatitis over time. **A** individual studies **B** pooled data for each decade (mean + SEM).

**Fig 3:**
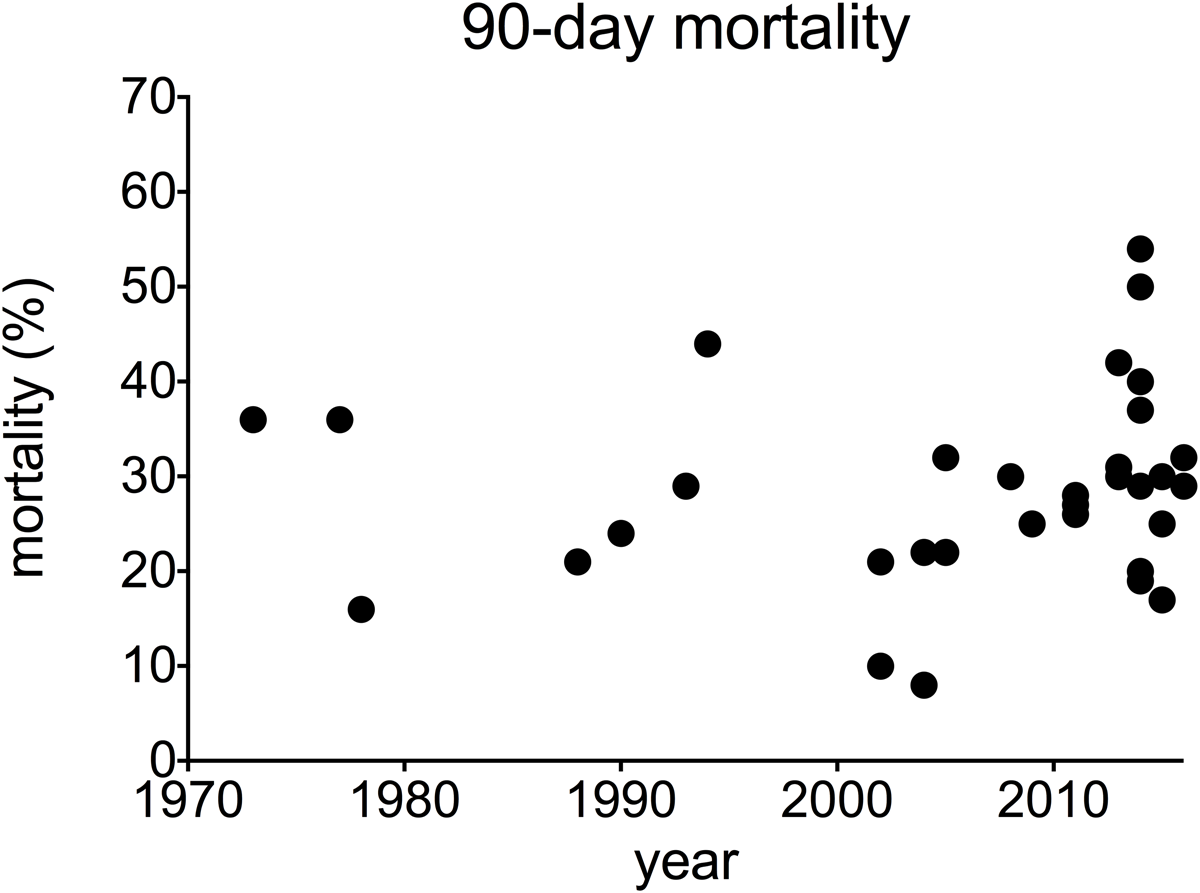
90-day mortality in alcoholic hepatitis over time. **A** individual studies **B** pooled data for each decade (mean + SEM).

**Fig 4:**
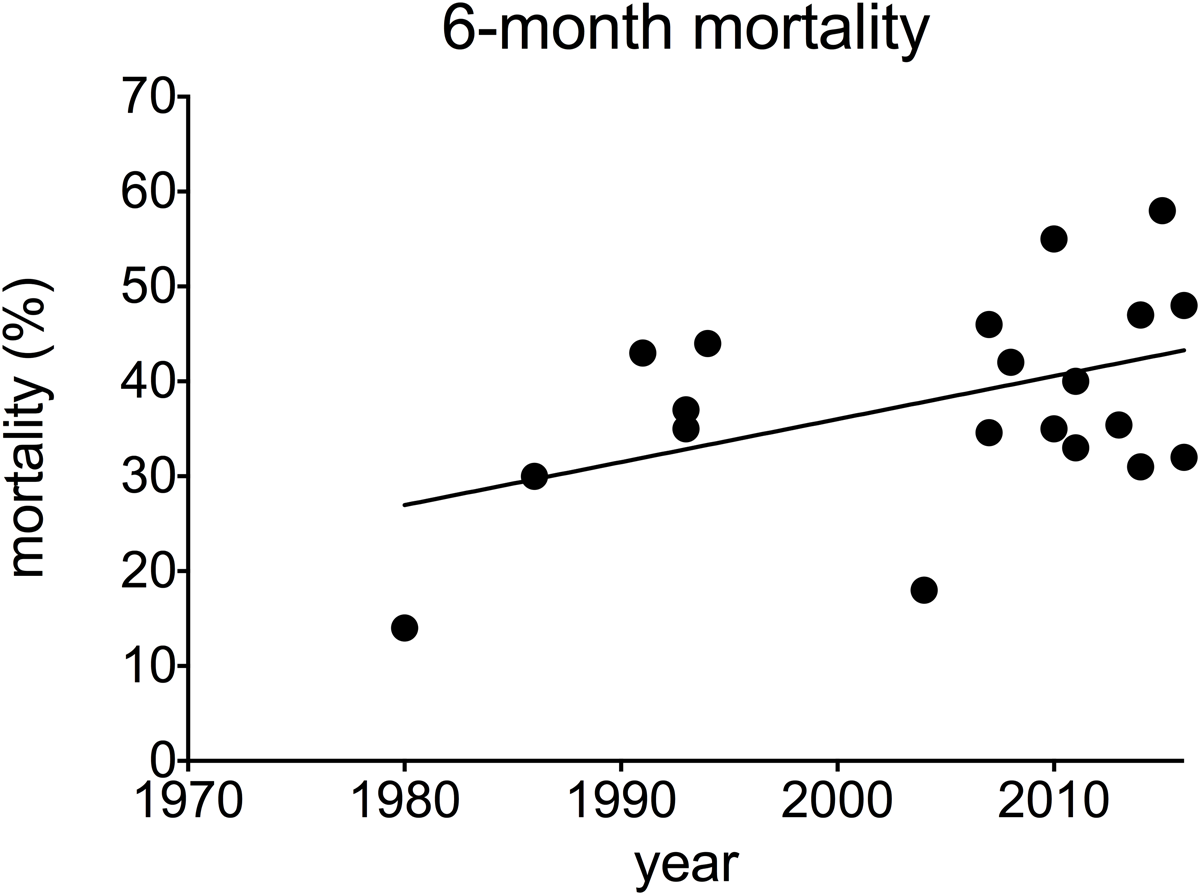
180-day mortality in alcoholic hepatitis over time. **A** individual studies **B** pooled data for each decade (mean + SEM).

When all studies where taken into consideration, average mortality from AH was 25% at 28 days after admission and 29% at 90 days after admission. This has not changed over time: univariable analysis of trends over time showed no significant changeover time in 28-day mortality (Pearson correlation -0.216, p = 0.098, data from 61 studies, 6652 participants) (Fig 2), or 90-day mortality (correlation 0.121 p = 0.503, 52 studies, 5672 participants) (Fig 3). Overall mortality at 180 days after admission was 38%. A small but statistically significant increase in 180-day mortality after admission was observed over time (correlation 0.461 p = 0.036, 21 studies, 2593 participants) (Fig 4). These findings are summarised in Table 1.

**Table 1:** Pearson correlation between year of study and time-point mortality in acute alcoholic hepatitis.

	28-day mortality	p	90-day mortality	p	180-day mortality	p
Overall	-0.216	0.098	0.121	0.503	0.461	0.036*
Sub-group analyses						
RCT	-0.305	0.055	0.152	0.619	0.455	0.187
RCT Intervention groups	-0.259	0.117	-0.80	0.814	0.502	0.169
RCT Control groups	-0.341	0.033*	-0.077	0.821	0.474	0.197
OS	0.276	0.214	0.200	0.475	-0.354	0.491
Biopsy proven	0.078	0.801	0.009	0.979	-0.499	0.208
Severe disease (mDF >32)	-0.130	0.419	0.342	0.95	0.110	0.707

*significant at p<0.05

### Meta-regression analysis

Creatinine, bilirubin, white cell count and age were associated with mortality (Table 2). Moreover these factors showed evidence of change over time. To analyse changes in mortality over time whilst controlling for these factors, linear meta-regression was performed. In this analysis, mortality did not significantly change over time at 28-days (beta -0.715, p = 0.058,), 90-days (beta 0.571, p = 0.341) or 180-days (beta 0.552, p = 0.381) after admission with AH (Table 3).

**Table 2:** Factors associated with mortality in alcoholic hepatitis and their change over time.

		Mortality			
		28-day mortality	90-day mortality	180-day mortality	Change over time
Prothrombin time seconds	Correlation	-0.093	-0.019	0.059	**0.295**
	p	0.565	0.938	0.856	**0.037**
Creatinine mg/dL	Correlation	**0.359**	0.184	0.314	**-0.358**
	p	**0.034**	0.464	0.321	**0.015**
Bilirubin mg/dL	Correlation	**0.496**	**0.655**	**0.597**	-0.134
	p	**<0.001**	**0.001**	**0.031**	0.307
White cell count x10^9^/L	Correlation	**0.561**	**0.515**	0.548	-0.497**
	p	**0.001**	**0.034**	0.127	**0.001**
Age years	Correlation	**-0.522**	-0.390	0.132	0.214
	p	**0.007**	0.135	0.700	0.223
Gender % male	Correlation	-0.077	-0.004	-0.255	-0.156
	p	0.776	0.989	0.626	0.477

**Table 3:** Meta-regression of mortality over time. Data are beta values from linear regression to control for other factors associated with mortality.

	28-day mortality	p	90-day mortality	p	180-day mortality	p
Overall	0.715	0.058	0.571	0.341	0.552	0.381
Sub-group analyses						
RCT control group	-0.538	0.231				

### Subgroup analyses

Included studies were grouped according to the type of study: therapeutic trials or observational studies. The frequency of biopsy proven diagnosis of AH, and severity of disease was also considered.

When considering therapeutic trials only, no statistically significant change in mortality over time was observed at each time point: 28-day mortality, Pearson correlation -0.305 p = 0.055, (40 studies, 3888 participants), 90-day mortality, correlation 0.152 p = 0.619 (13 studies, 2054 participants), 180-day mortality (correlation 0.455 p = 0.187, 10 studies, 1041 participants). When intervention and control groups were considered separately, a small but statistically significant improvement in 28-day mortality was seen in control groups (Pearson correlation -0.341, p-0.034)(Table 1). After controlling for other factors associated with mortality in meta-regression, this association was no longer significant (beta -0.538, p = 0.231). In observational studies, no changes in mortality were seen at 28-days (correlation 0.276 p = 0.214, 22 studies, 2958 participants), 90-days (correlation 0.200, p = 0.475, 20 studies, 2604 participants) or 180-days, (correlation -0.354, p = 0.491, 11 studies, 3581 participants)(Table 1).

When considering studies that only included biopsy-proven AH (16 studies, 2215 participants), no change in mortality over time was seen at any time point: 28-days (correlation 0.078 p = 0.801, 13 studies, 1459 participants), 90-days (correlation -0.009, p = 0.979, 10 studies, 970 participants) or 180-days, (correlation -0.499, p = 0.208, 8 studies, 1179 participants)(Table 1).

Finally, to eliminate non-severe alcoholic hepatitis we performed sub-group analysis including only those studies that had a mean discriminant function (DF) above 32. This excluded 13 studies that did not report an average DF, or data to allow the DF to be calculated, and 11 studies with a mean DF below 32. This left a group of 54 studies including 6503 participants. Once again, we saw no changes in mortality over time: in 28-day mortality (correlation -0.130 p = 0.419, 41 studies, 5268 participants), 90-days (correlation 0.342, p = 0.95, 25 studies, 4645 participants) or 180-days, (correlation -0.110, p = 0.707, 14 studies, 2473 participants)(Table 1).

## Discussion

Alcoholic hepatitis is an acute, life-threatening type of alcoholic liver disease. There has been ongoing research for many years in AH and many therapeutic agents have been examined for efficacy. However, our data show that mortality from AH has not improved over more than four decades. Indeed we found a small but significant increase in mortality at 180-days. This observation remained when considering only therapeutic trials where inclusion and exclusion criteria may have been more stringent. These results demonstrate the urgent need for better treatment in AH.

The strength of this analysis rests on the use of robust systematic review to obtain a comprehensive data set of studies in AH over the past few decades. Despite some missing data, particularly for outcomes beyond 28 days, this dataset was large enough to allow associations to be tested in univariable and multivariable analysis. In terms of limitations of the data, our conclusions depend on aggregate data reported in studies, where individual patient data may give more reliable results. However the difficulties in obtaining such data is considerable especially with older studies. The apparent increase in 6-month mortality is likely due to the small number of trials reporting this longer outcome in the 1970s. Information on cause of death would be valuable as this may have changed over time, however this was rarely reported.

Our data are consistent with previous work examining outcomes over time in liver disease. Analysis of survival of people with liver disease after discharge from hospitals in England from 1968–1999 found no improvement in 1-month or 1-year survival over time [24]. It is notable that the majority of this cohort had alcohol related liver disease, although interestingly the overall survival was much better for patients with AH compared to decompensated cirrhosis. Although there may be some methodological concerns over these data [25], given that there are no new specific treatments for ArLD that have emerged since these data were reported (in contrast to other causes of chronic liver disease) it is reasonable to think that the data remain relevant.

Whilst progress towards novel specific treatments in AH has been frustratingly slow, it might be expected that improvements in medical care of acutely unwell patients would have a positive effect on outcomes. Our results do not support this. In other areas of hepatology for example variceal bleeding or decompensated cirrhosis clear and standardised care bundles are used [26] that revolve around early assessment and administration of prophylactic antibiotics for example. Many patients with AH present to non-specialist medical services, where a standardised care bundle may allow for better treatment in the early stages of disease. Equally important is the ongoing pursuit of better treatments for AH that may make a significant impact on outcomes. This is especially pertinent given the underwhelming evidence for the efficacy of corticosteroids [15] which had been by many seen as central to the treatment of AH [27], despite conflicting trial data.

In conclusion, review of the available literature regarding mortality in alcoholic hepatitis over four decades shows no evidence of improvement in short-term mortality over time. This is in contrast to significant improvements in outcome in other liver diseases and other prevalent medical conditions.

## Supporting information

S1 Table Outcomes of included randomised clinical trials. (DOCX)

S2 Table Outcomes of included observational studies. (DOCX)

S3 Table Characteristics of patients in randomised clinical trials. (DOCX)

S4 Table Characteristics of patients in observational studies. (DOCX)

S5 Table Inclusion and exclusion criteria of included studies. (DOCX)

S6 Table Risk of bias in randomised Studies—Assessed with Cochrane risk of bias tool. (DOCX)

S7 Table Risk of bias in observational studies. (DOCX)

S8 Table Summary of missing data. (DOCX)

S1 Prism Statement (DOCX)

S1 References (DOCX)
