# Correction: Survival from alcoholic hepatitis has not improved over time

**DOI:** 10.1371/journal.pone.0195857

**Published:** 2018-04-10

**Authors:** Emily Hughes, Laurence J. Hopkins, Richard Parker

There are errors in the captions for Figs 2–4. Please see the correct captions here.

**Fig 2:**
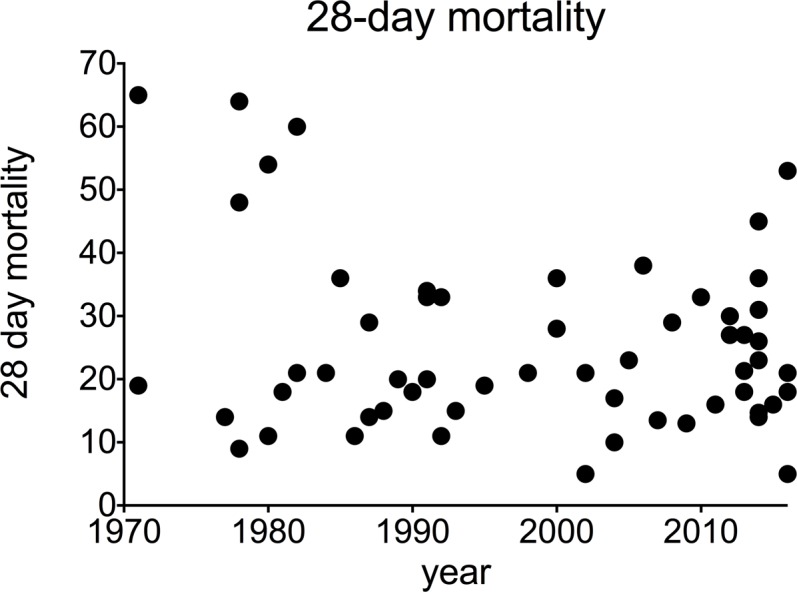
28-day mortality in alcoholic hepatitis over time. Each data point represents an individual study.

**Fig 3:**
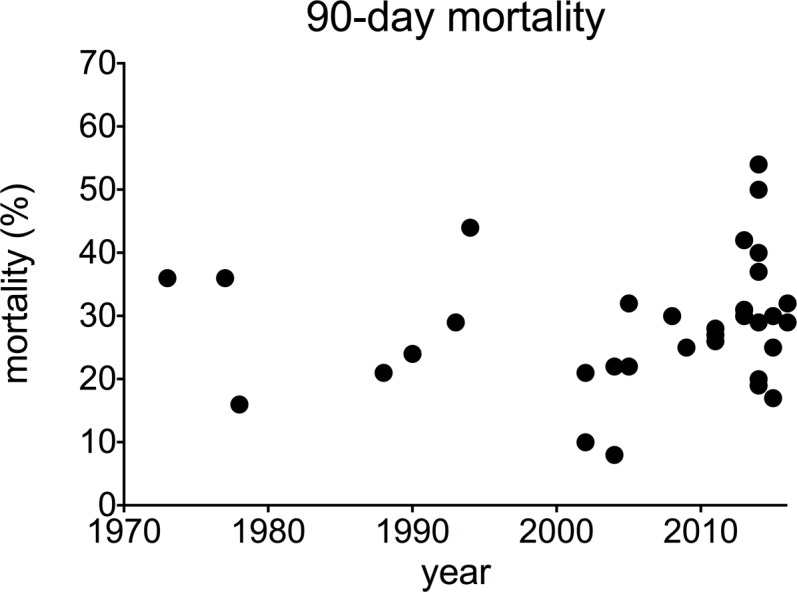
90-day mortality in alcoholic hepatitis over time. Each data point represents an individual study.

**Fig 4:**
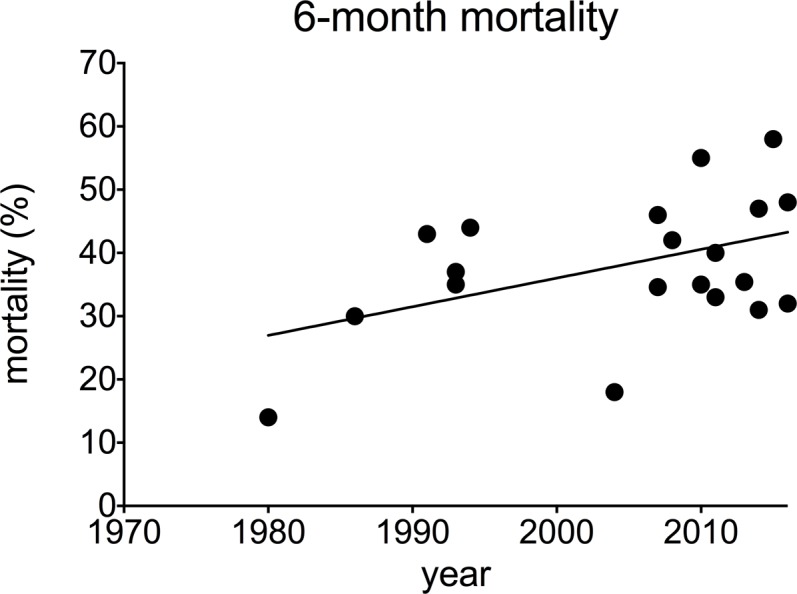
180-day mortality in alcoholic hepatitis over time. Each data point represents an individual study (correlation 0.461 p = 0.035).
