# Supplementary material for: Survival from alcoholic hepatitis has not improved over time
Source: PLoS One. 2018 Feb 14;13(2):e0192393. doi: 10.1371/journal.pone.0192393 (PMC5812634; doi:10.1371/journal.pone.0192393)
Supplement: S1 Table — (DOCX) [file pone.0192393.s001.docx]

Supplementary table 1: randomised clinical trials

| First author | Year | n | | | Intervention | Control | Duration | 28-day mortality | | | | | 90 day mortality | | | 180 day mortality | | | |
| --- | --- | --- | --- | --- | --- | --- | --- | --- | --- | --- | --- | --- | --- | --- | --- | --- | --- | --- | --- |
|  |  | All | Trial | Control |  |  |  | All | Trial | | Control | | All | Trial | Control | All | Trial | Control | |
| Helman (1) | 1971 | 37 | 20 | 17 | Prednisolone | Placebo | 28 days | 19% | 5% | | 35% | |  | | | | | | |
| Porter (2) | 1971 | 20 | 11 | 9 | Methylprednisolone | Placebo | 10 days | 65% | 55% | | 78% | |  | | | | | | |
| Campra (3) | 1973 | 45 | 20 | 25 | Prednisolone |  | 42 days |  | | | | | 36% | 36% | 35% |  | | | |
| Blitzer (4) | 1977 | 33 | 17 | 16 | Prednisolone | Placebo | 26 days | 14% | 17% | | 13% | | 36% | 50% | 31% |  | | | |
| Lesesne (5) | 1978 | 14 | 7 | 7 | Calorie supplements | Prednisolone | 30 days | 64% | 100% | | 29% | |  | | | | | | |
| Maddrey (6) | 1978 | 57 | 25 | 32 | Prednisolone | Placebo | 32 days | 9% | 4% | | 13% | | 16% | 13% | 19% |  | | | |
| Shumaker (7) | 1978 | 27 | 12 | 15 | Methylprednisolone | Placebo | 28 days | 48% | 50% | | 47% | |  | | | | | | |
| Depew (8) | 1980 | 28 | 15 | 13 | Prednisolone | Placebo | 28 days | 54% | 53% | | 54% | |  | | | | | | |
| Nasrallah (9) | 1980 | 35 | 18 | 17 | Amino acid | Placebo | 28 days | 11% | 0% | | 22% | |  | | | 14% | 10% | 20% | |
| Baker (10) | 1981 | 50 | 25 | 25 | Insulin and Glucagon | Placebo | 21 days | 18% | 12% | | 24% | |  | | | | | | |
| Hallé (11) | 1982 | 67 | 31 | 36 | Propylthiouracil | Placebo | 42 days | 21% | 23% | | 19% | |  | | | | | | |
| Theodossi (12) | 1982 | 55 | 27 | 28 | Methylprednisolone | Placebo | 3 days | 60% | 63% | | 57% | |  | | | | | | |
| Mendenhall (13) | 1984 | 263 | 90, 85 | 88 | Oxandrolone or Prednisolone | Placebo | 30 days | 21% |  | | | |  | | | | | | |
| Calvey (14) | 1985 | 64 | 42 | 22 | Protein supplementation | Control diet | 21 days | 36% | 38% | | | 32% |  | | | | | | |
| Achord (15) | 1987 | 28 | 14 | 14 | amino acid-glucose |  | 28 days | 14% | 7% | | | 21% |  | | | | | | |
| Feher (16) | 1987 | 66 | 33 | 33 | Insulin/Dextrose | Placebo | 21 days | 29% | 15% | | | 42% |  | | | | | | |
| Simon (17) | 1988 | 34 | 16 | 18 | Parenteral nutrition | Standard care | 28 days | **15%** | 40% | | | 25% | 21% |  | |  | | | |
| Carithers (18) | 1989 | 66 | 35 | 31 | Methylprednisolone | Placebo | 28 days | **20%** | 6% | | | 35% |  | | | | | | |
| Akriviadis (19) | 1990 | 72 | 36 | 36 | Colchicine | Placebo | 30 days | **18%** | 19% | | | 17% | 24% | 25% | 22% |  | | | |
| Mezey (20) | 1991 | 54 | 28 | 26 | Amino acid supplementation | Dextrose solution | 28 days | **20%** | 21% | | | 19% |  | | | | | | |
| Bird (21) | 1991 | 86 | 43 | 43 | Insulin and glucagon | Placebo | 3 weeks | **34%** | 35% | | | 33%33%ulin and glucagon tion(SMC) s are summaried in table 1. ent of alcoholic hepaitis.hepatitis over four decades, there is no e3333 |  | | | 43% | 50% | | 37% |
| Trinchet (22) | 1992 | 72 | 37 | 35 | Insulin and glucagon | glucose infusion | 3 weeks | **11%** | 27% | | | 14% |  | | | | | | |
| Ramond (23) | 1992 | 61 | 32 | 29 | Prednisolone | Placebo | 28 days | **33%** | 13% | | | 37% |  | | | | | | |
| Mendenhall (24) | 1993 | 273 | 137 | 136 | Oxandrolone + food supplement | Placebo | 28 days | **15%** | 9% | | | 21% | 29% | 20% | 37% | 37% | 35% | | 39% |
| Bird (25) | 1998 | 62 | 32 | 30 | Amlodipine | Placebo | 1 year | **21%** | 19% | | | 23% |  | | | | | | |
| Cabre (26) | 2000 | 71 | 36 | 35 | Enteral feeding | Prednisolone | 28 days | **28%** | 31% | | | 25% |  | | | | | | |
| Akriviadis (27) | 2000 | 101 | 49 | 52 | PTX | Placebo | 28 days | **36%** | 25% | | | 46% |  | | | | | | |
| Spahr (28) | 2002 | 20 | 11 | 9 | Infliximab | Placebo | 28 days | **5%** | 9% | | | 0% | 10% | 18% | 0% |  |  | |  |
| Mezey (29) | 2004 | 51 | 25 | 26 | Vitamin E | Placebo | 90 days |  | | | | | 8% | 8% | 8% | 18% | 16% | | 19% |
| Naveau (30) | 2004 | 36 | 18 | 18 | Infliximab + prednisolone | Prednisolone | 28 days | **17%** | | 28% | | 6% |  | | | | | | |
| Phillips (31) | 2006 | 101 | 48 | 53 | Antioxidants | Corticosteroid |  | **38%** | | 46% | | 30% |  | | | | | | |
| Stewart (32) | 2007 | 70 | 36 | 34 | Antioxidants | Placebo | 180 days |  | | | | | | | | 46% | 47% | | 44% |
| Boetticher (33) | 2008 | 48 | 26 | 22 | Etanercept | Placebo | 18 days | **29%** | | 35% | | 23% |  | | | 42% | 60% | | 20% |
| De (34) | 2009 | 68 | 34 | 34 | Pentoxifylline | Prednisolone | 28 days | **13%** | | 6% | | 21% | 25% | 15% | 35% |  | | | |
| Moreno (35) | 2010 | 47 | 27 | 20 | N-acetylcysteine. | Enteral nutrition | 14 days | **33%** | | 30% | | 16% |  |  |  | 35% | 40% | | 30% |
| Nguyen-Khac (36) | 2011 | 174 | 85 | 89 | Prednisolone + NAC | Prednisolone |  | **16%** | | 8% | | 24% | 28% | 22% | 34% | 33% | 27% | | 38% |
| Sidhu (37) | 2012 | 50 | 25 | 25 | Pentoxifylline | Placebo | 28 days | **30%** | | 20% | | 40% |  | | | | | | |
| Sidhu (38) | 2012 | 70 | 36 | 34 | Prednisolone + PTX | Prednisolone | 28 days | **27%** | | 28% | | 25% |  | | | | | | |
| Singh (39) | 2014 | 46 | 23 | 23 | GCSF | Standard care |  |  | | | | | 50% | 78% | 30% |  | | | |
| Higuera-de la Tijera (40) | 2014 | 78 | 39 | 39 | Metadoxine + Prednisolone | Standard care | 30 days | **36%** | | 26% | | 54% | 40% | 31% | 80% |  | | | |
| Park (41) | 2014 | 121 | 62 | 59 | Pentoxifylline | Prednisolone | 28 days | **14%** | | 24% | | 12% |  | | | 31% | 65% | | 73% |
| Thursz (42) | 2015 | 1103 | 827 | 276 | Pentoxifylline +/-Prednisolone | Placebo | 28 days | **16%** | | 16% | | 17% | 30% |  |  |  | | | |
| Moreno (43) | 2016 | 136 | 68 | 68.0 | Enteral nutrition | Control | 28 days | **18%** | | 16% | | 21% |  | | | 48% | 44% | | 52% |
| Tkachenko (44) | 2016 | 40 | 20 | 20.0 | Prednisolone + SAMe | Prednisolone | 28 days | **5%** | | 0% | | 10% |  | | | | | | |
