# Supplementary material for: Survival from alcoholic hepatitis has not improved over time
Source: PLoS One. 2018 Feb 14;13(2):e0192393. doi: 10.1371/journal.pone.0192393 (PMC5812634; doi:10.1371/journal.pone.0192393)
Supplement: S2 Table — (DOCX) [file pone.0192393.s002.docx]

Supplementary table 2: observational cohorts

| Title | Year | Country | n | **28-day mortality** | **90-day mortality** | **180-day mortality** |
| --- | --- | --- | --- | --- | --- | --- |
| Chedid (45) | 1986 | USA | 225 | 11% |  | 30% |
| Sheron (46) | 1991 | UK | 58 | 33% |  |  |
| Hill (47) | 1993 | USA | 50 |  |  | 35% |
| Fang (48) | 1994 | USA | 25 |  | 44% | 44% |
| Rodriguez-Rodriguez (49) | 1995 | Spain | 42 | 19% |  |  |
| Sheth (50) | 2002 | USA | 34 | **21%** | 21% |  |
| Spahr (51) | 2004 | Switzerland | 48 | **10%** | 22% |  |
| Cuthbert (52) | 2014 | USA | 148 | 23% | 37% | 47% |
| Dunn (53) | 2005 | USA | 73 |  | 22% |  |
| Forrest (54) | 2005 | UK | 241 | 23% | 32% |  |
| Louvet (55) | 2007 | France | 295 | 14% |  | 35% |
| Dominguez (56) | 2008 | Spain | 183 |  | 30% |  |
| di Mambro (57) | 2011 | UK | 20 |  |  | 55% |
| Sandahl (58) | 2011 | Denmark | 274 | 16% | 27% | 40% |
| Spahr (59) | 2011 | Switzerland | 163 |  | 26% |  |
| Pang (60) | 2015 | Canada | 122 |  | 17% |  |
| Sancho-Bru (61) | 2012 | Spain | 59 | 27% |  |  |
| Lafferty (62) | 2013 | UK | 182 | 18% | 31% |  |
| Potts (63) | 2013 | UK | 109 | 21% | 30% | 35% |
| Monsanto (64) | 2013 | Portugal | 45 | 27% | 42% |  |
| Altamirano (65) | 2014 | Spain | 121 |  | 29% |  |
| Papastergiou (66) | 2014 | UK | 71 | 14% | 20% |  |
| Goyal (67) | 2014 | India | 104 | 31% |  |  |
| Kadian (68) | 2014 | India | 47 | 26% |  |  |
| Mazzocco (69) | 2014 | UK | 82 | 45% |  |  |
| Rakachonda (70) | 2014 | USA | 76 |  |  | 47% |
| Lee (71) | 2014 | South Korea | 404 | 15% | 19% |  |
| Michelena (72) | 2015 | Multicenter | 162 |  | 25% |  |
| Gustot (73) | 2014 | Belgium | 92 |  | 54% |  |
| Serste (74) | 2015 | Europe | 139 |  |  | 58% |
| Andrade (75) | 2016 | Portugal | 34 |  | 29% |  |
| Ravi (76) | 2016 | USA | 105 | 53% |  |  |
| Beisel (77) | 2016 | Germany | 73 | 21% | 32% | 32% |
