# Supplementary material for: Survival from alcoholic hepatitis has not improved over time
Source: PLoS One. 2018 Feb 14;13(2):e0192393. doi: 10.1371/journal.pone.0192393 (PMC5812634; doi:10.1371/journal.pone.0192393)
Supplement: S3 Table — (DOCX) [file pone.0192393.s003.docx]

Supplementary table 3: characteristics of patients in randomised studies

| First author | Year | n | Intervention | PT  seconds | Urea  mmol/L | Creatinine  mg/dL | Bilirubin mg/dL | White cell count  x10^9^/L | Age  years | Alcohol intake  g/day | Gender  (% male) | Biopsy |
| --- | --- | --- | --- | --- | --- | --- | --- | --- | --- | --- | --- | --- |
| Helman (1) | 1971 | 37 | Prednisolone | 14.76 |  |  | 10.8 |  | 47.8 |  | 32% |  |
| Porter (2) | 1971 | 20 | Methylprednisolone |  |  |  | 20.9 | 18.4 |  |  |  | 100% |
| Campra (3) | 1973 | 45 | Prednisolone |  | 21.35 | 1.75 | 18.2 | 17.8 |  |  |  | 35% |
| Blitzer (4) | 1977 | 33 | Prednisolone |  |  |  | 20.4 |  |  |  | 100% | 49% |
| Lesesne (5) | 1978 | 14 | Calorie supplements |  | 30.05 | 2.00 | 23.0 | 15.6 |  |  |  | 18% |
| Maddrey (6) | 1978 | 57 | Prednisolone | 15.65 | 15.95 | 1.40 | 11.5 | 11.8 |  |  |  | 79% |
| Shumaker (7) | 1978 | 27 | Methylprednisolone |  |  |  |  |  |  |  |  | 44% |
| Depew (8) | 1980 | 28 | Prednisolone |  |  | 1.20 | 21.6 | 16.4 |  |  |  |  |
| Nasrallah (9) | 1980 | 35 | Amino acid |  |  |  | 9.8 |  | 46.3 |  |  | 4% |
| Baker (10) | 1981 | 50 | Insulin and Glucagon | 16.40 |  |  | 10.4 |  |  |  |  | 29% |
| Hallé (11) | 1982 | 67 | Propylthiouracil | 29.90 |  | 0.95 | 17.8 | 14.4 |  |  |  | 32% |
| Theodossi (12) | 1982 | 55 | Methylprednisolone | 10.50 |  | 1.21 | 14.4 | 13.0 |  |  |  | 42% |
| Mendenhall (13) | 1984 | 263 | Oxandrolone or Prednisolone | 4.03 | 18.33 | 1.60 | 15.5 | 11.5 |  |  |  | 91% |
| Calvey (14) | 1985 | 64 | Protein supplementation |  |  |  |  |  | 49.0 |  | 50% | 32% |
| Achord (15) | 1987 | 28 | amino acid-glucose |  |  |  |  |  |  |  |  | 77% |
| Feher (16) | 1987 | 66 | Insulin/Dextrose | 20.72 |  |  | 15.4 |  | 46.5 | 176 | 58% |  |
| Simon (17) | 1988 | 34 | Parenteral nutrition | 16.30 |  |  | 9.5 |  |  |  |  | 77% |
| Carithers (18) | 1989 | 66 | Methylprednisolone | 18.20 |  | 1.52 | 17.5 |  |  |  |  | 56% |
| Akriviadis (19) | 1990 | 72 | Colchicine |  |  | 1.10 | 17.4 | 16.3 |  |  | 67% |  |
| Mezey (20) | 1991 | 54 | Amino acid supplementation | 17.10 |  |  | 15.8 |  |  |  |  | 29% |
| Bird (21) | 1991 | 86 | Insulin and glucagon | 23.10 | 10.45 | 2.18 | 14.1 | 13.0 |  |  |  | 0% |
| Trinchet (22) | 1992 | 72 | Insulin and glucagon | 20.16 |  | 1.14 | 15.5 | 11.5 |  |  |  | 70% |
| Ramond (23) | 1992 | 61 | Prednisolone | 38.00 |  | 1.05 | 14.6 |  |  |  | 45% | 100% |
| Mendenhall (24) | 1993 | 273 | Oxandrolone + food supplement |  |  |  |  |  |  |  |  | 100% |
| Bird (25) | 1998 | 62 | Amlodipine | 19.80 | 5.40 | 1.01 | 8.3 |  | 52.4 |  | 60% |  |
| Cabre (26) | 2000 | 71 | Enteral feeding | 1.40 | 29.05 | 0.95 | 16.7 | 10.2 |  |  |  |  |
| Akriviadis (27) | 2000 | 101 | PTX | 19.70 |  | 1.25 | 19.5 | 16.5 | 41.6 |  | 70% | 52% |
| Spahr (28) | 2002 | 20 | Infliximab | 45.00 |  | 0.00 | 7.2 |  | 53.0 |  | 80% |  |
| Mezey (29) | 2004 | 51 | Vitamin E | 15.25 |  | 0.80 | 8.2 |  | 47.5 | 134 | 80% | 100% |
| Naveau (30) | 2004 | 36 | Infliximab + prednisolone | 37.50 |  | 0.82 | 14.4 |  | 52.0 | 111 | 69% | 10% |
| Phillips (31) | 2006 | 101 | Antioxidants | 21.91 |  | 1.17 | 26.3 | 14.6 |  |  |  | 100% |
| Stewart (32) | 2007 | 70 | Antioxidants | 25.75 |  | 1.29 | 25.0 | 13.8 | 44.3 |  | 54% | 64% |
| Boetticher (33) | 2008 | 48 | Etanercept | 23.10 | 0.00 | 1.40 | 19.7 |  |  |  | 73% | 63% |
| De (34) | 2009 | 68 | Pentoxifylline | 28.07 | 28.66 | 1.31 | 6.0 |  |  |  |  |  |
| Moreno (35) | 2010 | 47 | N-acetylcysteine. | 44.25 |  | 0.88 | 13.6 | 10.4 | 48.5 |  | 72% | 0% |
| Nguyen-Khac (36) | 2011 | 174 | Prednisolone + NAC | 19.38 | 0.00 | 0.83 | 14.7 | 10.7 | 52.8 | 107 |  | 100% |
| Sidhu (37) | 2012 | 50 | Pentoxifylline | 25.30 | 50.30 | 2.10 | 22.0 |  |  |  | 100% | 100% |
| Sidhu (38) | 2012 | 70 | Prednisolone + PTX |  |  |  | 20.2 |  |  |  |  | 0% |
| Singh (39) | 2014 | 46 | GCSF | 31.10 | 0.00 | 1.15 | 20.1 | 15.8 |  |  |  | 0% |
| Higuera-de la Tijera (40) | 2014 | 78 | Metadoxine + Prednisolone | 21.55 | 54.05 | 1.50 | 24.4 | 19.7 |  | 321 |  |  |
| Park (41) | 2014 | 121 | Pentoxifylline | 30.10 | 0.00 | 1.40 | 17.5 | 10.6 |  |  |  |  |
| Thursz (42) | 2015 | 1103 | Pentoxifylline +/-Prednisolone | 21.30 |  | 0.88 | 17.6 | 10.1 | 48.7 | 174 | 63% | 0% |
| Moreno (43) | 2016 | 136 | Enteral nutrition | 25.20 |  | 0.75 | 12.6 |  |  |  |  | 19% |
| Tkachenko (44) | 2016 | 40 | Prednisolone + SAMe | 23.20 | 0.00 | 1.03 | 12.8 | 13.2 | 46.9 | 88 | 80% | 100% |
