# Supplementary material for: Survival from alcoholic hepatitis has not improved over time
Source: PLoS One. 2018 Feb 14;13(2):e0192393. doi: 10.1371/journal.pone.0192393 (PMC5812634; doi:10.1371/journal.pone.0192393)
Supplement: S4 Table — (DOCX) [file pone.0192393.s004.docx]

Supplementary table 4

| Title | Year | n | PT  seconds | Urea mmol/L | Creatinine  mg/dL | Bilirubin  mg/dL | White cell count  X10^9^/L | Age  years | Average alcohol intake | % male | Biopsy proven |
| --- | --- | --- | --- | --- | --- | --- | --- | --- | --- | --- | --- |
| Chedid (45) | 1986 | 225 |  |  |  |  |  |  |  |  |  |
| Sheron (46) | 1991 | 58 |  |  |  |  |  |  |  |  |  |
| Hill (47) | 1993 | 50 |  |  |  |  |  |  |  |  |  |
| Fang (48) | 1994 | 25 |  |  |  |  |  |  |  |  |  |
| Rodriguez-Rodriguez (49) | 1995 | 42 |  |  |  |  |  |  |  |  |  |
| Sheth (50) | 2002 | 34 |  |  |  |  |  |  |  |  |  |
| Spahr (51) | 2004 | 48 |  |  |  |  |  |  |  |  |  |
| Cuthbert (52) | 2014 | 148 |  |  |  |  |  | 45 |  | 80 |  |
| Dunn (53) | 2005 | 73 |  |  |  |  |  |  |  |  |  |
| Forrest (54) | 2005 | 241 | 21.0 |  |  | 9.06 |  |  |  |  | 33% |
| Louvet (55) | 2007 | 295 | 19.5 |  | 0.80 | 12.35 | 10.80 | 49.7 | 120 | 50.5 | 100% |
| Dominguez (56) | 2008 | 183 | 26.6 |  | 0.80 | 14.75 | 11.00 | 49 | 100 | 69 | 100% |
| di Mambro (57) | 2011 | 20 |  |  |  |  |  |  |  | 70% | 100% |
| Sandahl (58) | 2011 | 274 |  |  |  |  |  |  |  |  |  |
| Spahr (59) | 2011 | 163 |  |  |  | 5.32 | 7.40 | 55 |  | 63% | 100% |
| Pang (60) | 2015 | 122 |  |  |  | 10.23 | 9.00 | 49 | 121 | 60 |  |
| Sancho-Bru (61) | 2012 | 59 | 22.4 |  | 0.80 | 10.00 | 8.00 | 51 | 100 | 70 | 100% |
| Lafferty (62) | 2013 | 182 | 26.0 | 5.40 | 1.08 | 12.98 | 10.50 | 49 |  |  |  |
| Potts (63) | 2013 | 109 | 24.5 | 3.30 | 0.70 | 12.98 | 10.35 | 49.5 | 158.85 | 63 | 11% |
| Monsanto (64) | 2013 | 45 | 29.4 |  | 1.00 | 16.80 | 11.10 | 45.7 |  | 64.4 | 18% |
| Altamirano (65) | 2014 | 121 | 22.4 |  | 0.80 | 9.70 | 8.90 | 49 | 100 | 67 | 100% |
| Papastergiou (66) | 2014 | 71 | 22.2 |  |  | 12.40 | 11.20 | 49 |  | 66.2 | 100% |
| Goyal (67) | 2014 | 104 | 19.6 |  | 1.40 | 9.60 |  | 44.8 |  |  | 0% |
| Kadian (68) | 2014 | 47 | 29.4 |  |  | 6.02 | 11.60 | 47.4 |  | 100 |  |
| Mazzocco (69) | 2014 | 82 |  |  |  |  |  |  |  |  |  |
| Rakachonda (70) | 2014 | 76 | 28.0 |  | 1.00 | 15.20 | 11.10 | 49 |  | 55 | 4% |
| Lee (71) | 2014 | 404 | 23.8 |  | 1.00 | 6.00 | 9.20 | 54 | 113 | 84 |  |
| Michelena (72) | 2015 | 162 | 23.8 |  | 1.10 | 12.00 |  | 50 |  | 71 | 100% |
| Gustot (73) | 2014 | 92 | 19.1 |  | 0.90 | 15.05 |  |  |  |  |  |
| Serste (74) | 2015 | 139 | 19.0 |  | 1.28 | 17.10 |  | 52.2 |  | 64.7 |  |
| Andrade (75) | 2016 | 34 |  |  |  |  |  |  |  |  |  |
| Ravi (76) | 2016 | 105 |  |  |  |  |  |  |  |  |  |
| Beisel (77) | 2016 | 73 |  |  |  |  | 12.60 | 51 |  |  | 68% |
