# Supplementary material for: Survival from alcoholic hepatitis has not improved over time
Source: PLoS One. 2018 Feb 14;13(2):e0192393. doi: 10.1371/journal.pone.0192393 (PMC5812634; doi:10.1371/journal.pone.0192393)
Supplement: S5 Table — (DOCX) [file pone.0192393.s005.docx]

Supplementary table 5: Inclusion and Exclusion Criteria

|  | **Inclusion criteria** | **Exclusion criteria** |
| --- | --- | --- |
| Helman 1971 | Not described | Not described |
| Porter 1971 | Not described | Not described |
| Campra 1973 | Not described | Not described |
| Blitzer 1977 | Not described | Not described |
| Lesesne 1978 | Not described | Not described |
| Maddrey 1978 | Not described | Not described |
| Shumaker 1978 | Not described | Not described |
| Depew 1980 | Not described | Not described |
| Nasrallah & Galambos 1980 | Not described | Not described |
| Baker 1981 | Not described | Not described |
| Hallé 1982 | recent heavy ethanol ingestion and clinical diagnosis of alcoholic liver disease: serum bilirubin >5 mg/dl and one of: hepatic tenderness, fever above 100°F, or leukocytosis above 12,000 mm^3^ | Serious bacterial infection, massive gastrointestinal bleeding, pre-existing renal failure, previous or current thyroid disease |
| Theodossi 1982 | alcohol intake of 80 g or more daily for at least five years, serum bilirubin concentration greater than 80pmol/l, AST at least twice the upper limit of normal, prothrombin time prolonged by at least nine seconds | hepatoma, other diseases such as recent myocardial infarction, cerebrovascular accident |
| Mendenhall 1984 | conventional clinic and laboratory changes characteristic of AH | concominant conditions - Hep B surface antigen; evidence of parenteral drug abuse; intractable congestive heart failure, neoplasms that typically metastasise to liver, non-alcoholic causes of liver disease. Any conditions that contraindicated steroid therapy - infection, severe PUD, insulin dependent diabetes mellitus |
| Calvey 1985 | clinical and biochemical evidence of hepatocellular damage, alcohol intake greater than 80 g daily for several years continuing up to the onset of symptoms, poor hepatic uptake of 99mTcsulphur colloid on scinti-scanning | absence of serological evidence of acute hepatitis type A or B |
| Achord 1987 | None | None |
| Feher 1987 | None | None |
| Simon 1998 | history of chronic ethanol ingestion (>80 g/day for at least 2 years) and right lobe hepatomegaly. If biopsy could not be performed then following criteria had to be met: actively consuming ethanol up to admission, serum AST < 350 U/l, AST to ALT ratio > 2. If liver biopsy performed after randomization was not consistent with alcoholic hepatitis, the patient was withdrawn from the study. | acute pancreatitis, insulin-dependent diabetes mellitus, positive HBsAg, malignancy, hypotension, congestive heart failure, sepsis, severe chronic obstructive pulmonary disease, and recent severe trauma or surgery. |
| Carithers 1989 | AH and hepatic encephalopathy or a DF > 32 |  |
| Akriviadis 1990 | palpable hepatomegaly, serum bilirubin >5 mg/dL, and one or more of the following: hepatic tenderness, fever above l00F, and leukocytosis above 12,000/mm^3^ | life-threatening bacterial infection, massive gastrointestinal hemorrhage, renal insufficiency (creatinine > 2.5 mg/dL), HBV infection |
| Trinchet 1992 | biopsy-proven severe alcoholic hepatitis |  |
| Ramond 1992 | long standing history of alcoholism and clinical features of alcoholic hepatitis | GI bleeding or bacterial infection within the previous 2 days |
| Mendenhall 1993 | heavy alcohol intake and the presence of laboratory changes characteristic of alcoholic liver injury | nonalcoholic liver disease, severe congestive heart failure, insulin-dependent diabetes mellitus, prior bypass surgery, malignancy, recent parental drug abuse, HBV, HIV, inability or unwillingness to take oral medications or the food supplement and attend follow-up clinic, identification beyond the fifteenth day of hospitalization, participation in other therapeutic studies and female gender. |
| Bird 1998 | alcohol intake greater than 40 g/day in women and 60 g/day in men, and at least two of: palpable hepatomegaly, leukocytosis and typical appearances of liver and spleen on isotope scanning | pancreatitis, severe gastrointestinal haemorrhage, malignant disease or seropositivity for HBsAg, anti-HCV or anti-HIV.  Admission primarily for the control of complications of cirrhosis (bleeding oesophageal or gastric varices, hepatic encephalopathy, ascites or oedema) or previous admissions for the complications of portal hypertension |
| Akriviadis 2000 | jaundice, DF >32, and 1 or more of: palpable tender hepatomegaly, fever, leucocytosis, hepatic encephalopathy, and hepatic systolic bruit. | concomitant bacterial infections, active gastrointestinal haemorrhage, severe cardiovascular or pulmonary disease |
| Spahr 2002 | significant alcohol intake (100–150 g/day); liver biochemistry suggestive of AH, DF 32 - 55 | Renal failure, uncontrolled infection or recent gastrointestinal bleeding, DF>55. Presence of hepatitis B, C or HIV |
| Mezey 2004 | Aged 18–70 years, recent history of heavy alcohol ingestion, moderate elevation of AST, AST/ALT ratio >1.0, no evidence of liver disease due to viral hepatitis, autoimmune disease, hemochromatosis, Wilson’s disease or drug-induced hepatitis | pregnancy, breast feeding, cardiovascular, pulmonary or kidney disease, pancreatitis, type I diabetes, recent gastrointestinal bleeding, peptic ulcer disease, concurrent infection, thrombophlebitis, HIV positivity or history of ingestion of more than 100 I.U. vitamin E for the prior month |
| Naveau 2004 | Biopsy-proven AH | presence of hepatitis B surface antigen, hepatitis C virus or HIV antibodies, hepatocellular carcinoma, ethanol abstinence for more than 1 month, concomitant infection, severe bacterial infection within previous 3 months, concomitant or previous history of tuberculosis, severe associated disease (cardiac failure, severe pulmonary disease, neoplastic disease, severe psychiatric disorders), acute pancreatitis, gastrointestinal bleeding over the previous month, hepatorenal syndrome, or acanthocytosis |
| Phillips 2006 | history of heavy alcohol consumption (greater than 80 g alcohol per day for men, or greater than 60 g alcohol per day for women), prior to the onset of illness of at least 1-month duration, and absence of alternative aetiology of liver disease, serum bilirubin >100 μmol/L, serum AST <300 IU/L, serum IgA >5 g/L, white cell count >20×10^9^/L, ultrasound evidence of hepatic fatty infiltration, hepatomegaly | Active sepsis, active significant gastrointestinal haemorrhage within the previous 48 h, shock necessitating inotropic support, evidence of coexisting non-alcoholic liver disease, pregnant or lactating women. patients with a history of allergy to any component of the regimen. revious randomisation to the study, patients clearly improving spontaneously prior to trial entry. |
| Stewart 2007 | alcoholic hepatitis with hepatic encephalopathy and/or DF>32, recent heavy (>40 g/day for women; >60 g/day for men) alcohol intake, age between 18 and 65 and either a diagnostic liver biopsy or two of: hepatomegaly, leukocytosis and ‘‘white out’’ on liver and spleen isotope scanning | evidence of malignancy, positive HBV or HCV serology, pregnant or lactating women and cirrhotic patients admitted primarily for control of complications of portal hypertension |
| Boetticher 2008 | 18 years of age at entry with clinical evaluation and testing supporting a diagnosis of alcoholic hepatitis including jaundice, hepatomegaly, leukocytosis, fever, and elevations in transaminase levels, in the setting of compatible alcohol consumption.  Liver biopsy confirmation | hypersensitivity to etanercept, presence of infection including pneumonitis or sepsis or history of autoimmune disease, treatment with corticosteroids, pentoxifylline, propylthiouracil, or thalidomide in preceding 4 weeks prior to evaluation. In women, a negative pregnancy test, surgical sterility, or post menopausal state was a requirement for enrollment, breast-feeding women were not eligible |
| De 2009 | history of chronic alcohol intake of more than 50 g/day with clinical and biochemical features of severe alcoholic hepatitis (DF score ≥32 and AST:ALT >2:1 with absolute value of AST < 500 I.U/L and ALT < 200 IU/L) were included | other potential aetiology of liver injury even in the background of chronic alcohol intake |
| Moreno 2010 | biopsy proven alcoholic hepatitis (defined by the presence of satellitosis) and DF >32 at baseline | neoplastic disease compromising 6-month survival, positive HIV serology, and hepatorenal syndrome at randomization |
| Nguyen-Khac 2011 | Age 18 years or older, an average alcohol intake of more than 50g per day during the three months before enrollment, DF ≥32, liver histologic findings consistent with alcoholic hepatitis | Hepatorenal syndrome, hepatocellular carcinoma, uncontrolled bacterial infection or gastrointestinal haemorrhage in the previous 4 days, infection with HCV, HBV or HIV infection, autoimmune hepatitis, hemochromatosis, Wilson's disease, alpha-1 antitrypsin deficiency, acetaminophen induced hepatitis, cancer, N-acetyl-cysteine allergy, and serious cardiac, respiratory, or neurologic disease. |
| Sidhu 2012 | clinical features suggestive of alcoholic hepatitis (history of chronic alcoholism, recent onset of jaundice, fever, tender hepatomegaly), Patients with SAH (DF >32) were included | positive viral serology, concomitant infections, active GI haemorrhage, severe cardiovascular or pulmonary diseases, and a decline in DF to |
| Sidhu 2012b | AH and DF≥32 | active infection, bleeding, renal failure, or pancreatitis |
| Higuera-de la Tijera 2014 | Patients between 18 and 65 years old with clinical and biochemical criteria for SAH - heavy alcohol intake, rapid onset of jaundice in absence of biliary tract obstruction | acquired immunodeficiency syndrome, neoplasms, autoimmune diseases, psychiatric disorders different from alcoholism, history of atopy or asthma, diabetes, obesity, pregnancy, HBV or HCV infection, tuberculosis. any patients with intake of illict drugs, herbal products, antioxidant supplements, or previous treatment with steroids or pentoxifylline within the previous two years |
| Park et al 2014 | age of 20–75 years; an average alcohol intake of more than 40 g per day during the 3 months before enrolment; clinical features supporting a diagnosis of alcoholic hepatitis, including recent onset of jaundice in the prior 3 months as a prerequisite and at least one among common of hepatic encephalopathy, ascites, tender hepatomegaly, leukocytosis with predominantly neutrophilic differentiation, fever, or elevated liver enzymes; and DF≥32 | bacterial infection, gastrointestinal bleeding, concomitant viral hepatitis, renal impairment, pancreatitis, hepatocellular carcinoma, and other potential aetiologies of liver injury (autoimmune liver disease, Wilson’s disease, haemochromatosis, or drug-induced liver injury) |
| Thursz 2015 | age of 18 years or older, a clinical diagnosis of alcoholic hepatitis, an average alcohol consumption of more than 80 g per day for men and more than 60 g per day for women, a serum bilirubin level greater than 80 μmol per liter and a DF≥ 32 | jaundice for more than 3 months, cessation of alcohol consumption fro more than two months before randomisation, other causes of liver disease, AST > 500 IU per litre or ALT > 300 IU per litre, previous entry into the study within preceeding 6 months |
| Moreno 2016 | aged 18 to 75 years, were chronic alcohol consumers (>40 g/day), had a recent onset of jaundice within the past 3 months, biopsy-proven alcoholic hepatitis, DF ≥32 | presence of HBV surface antigen, HIV antibodies, chronic HCV infection, severe concurrent disease that compromised 6-month survival, uncontrolled infection, uncontrolled gastrointestinal bleeding, 1 hepatorenal syndrome, history of bariatric surgery, pentoxifylline, or molecular adsorbent recirculating system therapy |
| Tkachenko 2016 | age of 18 years or older, DF>32, average alcohol intake of more than 50 g/day during the 3 months before enrollment, and screening tests results (AUDIT ≥ 8 points and CAGE questionnare ≥ 3 points) | cessation of alcohol consumption for more than 2 months before randomization, SAMe, UDCA or pentoxifylline administration prior to hospitalization, presence of other causes of liver disease, uncontrolled infection or gastrointestinal haemorrhage, cancer, psychiatric disease, drug abuse, serious cardiac, respiratory, or neurologic disease, previous entry into the study within the preceding 6 months |
| Chedid | AH using serum bilirubin and prothrombin time to stratify | None |
| Sheron 1991 | history of alcohol intake exceeding 80g/day for I year, AST > 80 IU, hepatomegaly | None |
| Hill 1993 | male; moderate-to-severe alcoholic hepatitis | None |
| Rodriguez-Rodriguez 1995 | heavy drinkers of alcoholic beverages for more than 5 and acute AH: serum bilirubin > 4 mg/dl plus tender hepatomegaly or fever or leukocytosis over 12.000/mm | massive gastrointestinal hemorrhage, neoplasia, heart or renal failure, or with anti-human immunodeficiency virus antibodies |
| Sheth 2002 | alcoholic hepatitis was confirmed on clinical, biochemical and histological grounds | None |
| Spahr 2004 | excessive alcohol intake | serology for viral hepatitis and had no evidence of hepatocellular carcinoma. The absence of a severe infection associated to AH was verified by systematic ascitic fluid analysis and blood cultures in cases of fever |
| Cuthbert 2014 | jaundice from an acute decompensation in ALD (bilirubin > 5 mg/dL unaccounted for by another etiology or transfusion, AST increased and < 500 U/L with AST > ALT) | concomitant liver disease, persistent hyperbilirubinemia for > 2 months prior to admission, abstinence confirmed on multiple encounters, an alternative diagnosis or a previous index admission |
| Dunn | alcohol consumption within 2 months and exceeding 40 g/d for male and 20 g/d for female patients; AST/ALT > 1.5 with AST> 45 U/L; total bilirubin level above 2 mg/dL | absence of an alternative primary cause of liver disease based on clinical history and serological studies (11 patients with AH had underlying viral hepatitis but were not excluded because the clinical basis of the admission/visit was due to AH) |
| Forrest 2005 | serum bilirubin level ⩾80 μmol/l and a history of recent alcohol excess | viral hepatitis, autoimmune liver disease, or hepatocellular carcinoma |
| Louvet 2007 | history of alcoholism; liver chemistry suggestive of AH; transjugular liver biopsy | ≥active peptic ulcers, neoplasms, positive test for hepatitis B surface antigen, and human immunodeficiency virus antibodies |
| Dominguez | biopsy-proven AH | No access |
| di Mambro | decompensated chronic liver disease, DF >32, history of excess alcohol consumption | overt signs of sepsis or serum creatinine levels >400 μmol/L; nonalcohol-related liver disease, current or recent treatment (in the last 3 months) with oral or intravenous steroids or other immunosuppressants, documented HIV infection, or autoimmune disease. |
| Sandahl | initial discharge diagnosis of alcoholic hepatitis | None |
| Spahr | heavy recent alcohol intake (> 80 g/day) | radiological evidence of bile duct alterations, no documented hepatocellular carcinoma, no positive serology for hepatitis A, B, C or HIV, no disease associated with iron overload and no documented infection at the time of hospital admission and of liver biopsy (SBP excluded) |
| Pang | heavy alcohol consumption (>196 g/week or >56 g in any day among men, and >98 g/week or >42 g in any day among women) (24); elevated AST and/or ALT concentration, but <300 IU/mL, serum bilirubin >34 μmol/L; elevated INR | other causes of acute hepatic dysfunction |
| Sancho-Bru | clinical and analytical criteria of AH | did not fulfill all diagnostic criteria of AH, hepatocellular carcinoma |
| Lafferty 2013 | clinical history of excessive alcohol consumption (>80 g/day until at least 3 weeks before admission), supportive biochemical and clinical picture (new onset jaundice: serum bilirubin >80 μmol/L, AST and ALT <500 IU); GAHS ≥9 | acute drug toxicity, autoimmune liver disease, acute viral hepatitis, biliary obstruction or hepatocellular carcinoma; acute upper gastro-intestinal haemorrhage or with chronic viral hepatitis |
| Potts | jaundice (bilirubin >80 μmol/L) and coagulopathy (INR ≥1.5) with admission DF ≥32. Long-standing alcohol excess (>50 g/day in women and >70 g/day in men until at least 6 weeks prior to admission) and any of the following: AST:ALT ratio >2; Enlarged, tender liver with peripheral blood leukocytosis; Presence of hepatic encephalopathy at presentation  Corroborative liver biopsy, where available. | any other form of coexistent chronic liver disease (viral hepatitis, iron overload, biliary or autoimmune disease) or history of jaundice ≥3 months were excluded |
| Monsanto | total bilirubin >1.5 mg/dL, AST/ALT > 1.5 with an AST level above 75 IU/L, history of longstanding alcoholism (results available within first 24 hours) | absence of a coexistent primary cause of liver disease, namely viral hepatitis |
| Altamirano 2014 | excessive alcohol consumption (>60 g/day) prior to admission, moderately elevated aminotransferases with AST > ALT, high GGT and serum bilirubin levels, and a histological diagnosis of AH characterized by the presence of hepatocellular damage (hepatocellular ballooning and presence of Mallory bodies), inflammatory infiltrate (predominantly polymorphonuclear cells), and pericellular fibrosis | other causes of liver disease (concomitant hepatitis C virus n=16, concomitant hepatitis C virus/HIV co-infection n=3, drug-induced hepatotoxicity n=4, hemochromatosis n=1, military tuberculosis n=1 and syphilitic hepatitis n=1) or incomplete histological criteria of AH (n=34) |
| Papastergiou | AH on liver biopsy, history of alcohol abuse within the last 2 months (>40 g/day compatible with or men; >20 g/day for women), total serum bilirubin exceeding 2× ULN, AST:ALT >1.5 with AST 45 U/L | concomitant primary cause of liver disease. Patients with pre-existing viral hepatitis (n = 9) were not excluded |
| Goyal 2014 | Admission diagnosis of AH based on clinical and biochemical data | Positive viral markers or alternate diagnosis for liver dysfunction, HCC, portal vein thrombosis (PVT), significant comorbidities, extra hepatic biliary obstruction |
| Kadian | history of significant alcohol intake (> 40 g/day for women and > 60 g/day for men) for at least 10 years with recent excessive alcohol ingestion and clinical criteria including jaundice, hepatomegaly, fever, leucocytosis, and hyperbilirubinemia accompanied by modest increase in alkaline phosphatase level, AST and ALT elevated twofold to sevenfold (AST > ALT), AST/ALT ratio > 1, liver histology from liver biopsy where required | Other causes of hepatitis, including viral, autoimmune, non-alcoholic steatohepatitis, drugs, or metabolic disorders. Patients with diabetes mellitus, renal failure, alcohol withdrawal, stroke, pancreatitis, and malignant liver disease. |
| Mazzocco | history of excessive intake of alcohol over a period of several years and acute AH | co-existing viral hepatitis, auto-immune hepatitis, hepatocellular carcinoma or biliary obstruction. In addition, patients who died within 28 days of admission due to non-hepatic complications were also excluded from the study. |
| Rakachonda | age ≥18 years, history of chronic, heavy alcohol abuse, total bilirubin level of ≥3 mg/dl with AST>ALT and both <500 U/l, presence of either hepatomegaly or steatosis on imaging | abstinence from alcohol for at more than 6 weeks prior to admission |
| Lee | alcohol consumption within 2 months and exceeding 60 g/day for males and 40 g/day for females, rapid deterioration of liver function during the past 2 months, AST/ALT > 2, AST >45 IU/L and < 300 IU/L, and total bilirubin level > 2 mg/dL | liver disease causes other than alcohol consumption or HCC; those who died from non-liver-related causes, such as non-liver malignancies, cardiovascular events, or unknown reasons; those who exhibited uncontrolled infection or recent gastrointestinal bleeding within 15 days; and those receiving pentoxifylline and/or corticosteroids |
| Michelena 2015 | Histologically confirmed AH | Hepatocellular carcinoma or any other suspected cause of liver disease |
| Gustot | biopsy-proven diagnosis of severe AH and recent onset of jaundice (total bilirubin ⩾5 mg/dl) | active neoplasms associated with high short-term mortality (3 months), HBV surface antigen and HIV seropositivity, or positive HCV RNA |
| Serste | clinical and biological suspicion of AH and cirrhosis, age of at least 18 years, heavy consumption of alcohol (above 40 g/day for women and 50 g/day for men), DF ≥ 32, recent onset of jaundice within the past 3 months.  Histological proof of AH was mandatory | HBV surface antigen, HCV, HIV, transjugular intrahepatic porto-sytemic shunt, absence of pregnancy and hepatocellular carcinoma |
| Andrade | age > 18 years; excessive alcohol consumption (alcohol consumption >80 g/day for men and >60 g/day for women), moderate elevation (5–10 times upper limit of normal) of transaminases with AST > ALT, high GGT and bilirubin levels, absence of additional aetiology for liver disease, histologic diagnosis of AH | absence of additional aetiology for liver disease' |
| Ravi | Discharge diagnosis of alcoholic hepatitis | Bilirubin <5mg/dl, other causes of hepatitis, no recent alcohol use |
