# Supplementary material for: Survival from alcoholic hepatitis has not improved over time
Source: PLoS One. 2018 Feb 14;13(2):e0192393. doi: 10.1371/journal.pone.0192393 (PMC5812634; doi:10.1371/journal.pone.0192393)
Supplement: S6 Table — (DOCX) [file pone.0192393.s006.docx]

Supplementary table 6: Risk of bias in Randomised Studies – assessed with Cochrane Risk of Bias tool

|  | Sequence Generation | Allocation Concealment | Personnel Blinding | Outcome Assessor Blinding | Incomplete data addressed | Selective outcome reporting | Other bias |
| --- | --- | --- | --- | --- | --- | --- | --- |
| Helman 1971 (1) | U | U | N | Y | Y | N | N |
| Porter 1971 (2) | Y | Y | Y | Y | Y | N | N |
| Campra 1973 (3) | Y | Y | N | N | N | Y | Y |
| Blitzer 1977 (4) | Y | Y | Y | U | N | Y | N |
| Lesesne 1978 (5) | U | U | N | N | N | Y | N |
| Maddrey 1978 (6) | Y | Y | Y | Y | N | Y | N |
| Shumaker 1978 (7) |  |  |  |  |  |  |  |
| Depew 1980 (8) | Y | Y | Y | Y | N | N | Y |
| Nasrallah 1980 (9) | Y | Y | N | N | Y | Y | Y |
| Baker 1981 (10) | Y | Y | Y | Y | N | Y | Y |
| Hallé 1982 (11) | Y | Y | Y | Y | Y | Y | Y |
| Theodossi 1982 (12) | Y | Y | N | N | Y | Y | Y |
| Calvey 1985 (14) | Y | Y | N | N | N | N | N |
| Achord 1987 (15) |  |  |  |  |  |  |  |
| Feher 1987 (16) | U | Y | N | N | Y | Y | Y |
| Simon 1988 (17) | Y | Y | N | N | Y | Y | Y |
| Carithers 1989 (18) | Y | Y | Y | Y | Y | Y | Y |
| Trinchet.1992 (22) | U | U | Y | Y | N | N | N |
| Akriviadis 1990(19) | Y | Y | Y | Y | N | Y | Y |
| Mezey 1991 (20) | U | Y | Y | Y | Y | Y | Y |
| Bird 1991 (21) | Y | Y | Y | Y | Y | Y | Y |
| Ramond 1992 (23) | U | Y | Y | Y | Y | Y | U |
| Mendenhall 1993 (24) | Y | Y | N | Y | Y | Y | N |
| Bird 1998 (25) | Y | Y | Y | Y | Y | Y | Y |
| Cabre 2000 (26) | Y | Y | N | N | Y | Y | Y |
| Akriviadis 2000 (27) | Y | Y | Y | Y | Y | Y | Y |
| Spahr 2002 (28) | Y | Y | Y | Y | Y | Y | Y |
| Mezey 2004 (29) | Y | Y | Y | Y | Y | Y | Y |
| Naveau 2004 (30) | Y | Y | Y | Y | Y | Y | Y |
| Phillips 2006 (31) | Y | Y | N | N | Y | Y | Y |
| Stewart 2007 (32) | Y | Y | Y | Y | Y | Y | Y |
| Boetticher 2008 (33) | Y | Y | Y | Y | Y | Y | Y |
| De 2009 (34) | Y | Y | N | N | Y | Y | Y |
| Moreno 2010 (35) | Y | Y | Y | Y | Y | Y | Y |
| Nguyen-Khac 2011 (36) | Y | Y | N | N | Y | Y | Y |
| Sidhu (37) | Y | Y | N | N | Y | U | Y |
| Singh (39) | Y | Y | N | N | Y | Y | Y |
| Higuera-de la Tijera 2014 (40) | Y | Y | N | N | Y | Y | Y |
| Park 2014 (41) | Y | Y | N | N | Y | Y | Y |
| Thursz 2015 (42) | Y | Y | Y | Y | Y | Y | Y |
