# Supplementary material for: Survival from alcoholic hepatitis has not improved over time
Source: PLoS One. 2018 Feb 14;13(2):e0192393. doi: 10.1371/journal.pone.0192393 (PMC5812634; doi:10.1371/journal.pone.0192393)
Supplement: S8 Table — (DOCX) [file pone.0192393.s008.docx]

Supplementary table 8: missing data

|  |  | 28-day mortality | 90-day mortality | 180-day  mortality | Prothrombin time | Creatinine | Bilirubin | Gender | Alcohol intake |
| --- | --- | --- | --- | --- | --- | --- | --- | --- | --- |
| Trials | number of studies  (%) | 4 | 31 | 34 | 12 | 14 | 4 | 5 | 35 |
|  |  | 9% | 70% | 70% | 25% | 29% | 8% | 10% | 73% |
|  | number of participants (%) | 122 | 2054 | 3059 | 709 | 811 | 392 | 625 | 2420 |
|  |  | 3% | 50% | 25% | 17% | 20% | 10% | 15% | 59% |
| Observational studies | number of studies  (%) | 14 | 14 | 23 | 17 | 19 | 15 | 16 | 28 |
|  |  | 41% | 41% | 68% | 49% | 54% | 43% | 46% | 80% |
|  | number of participants (%) | 1260 | 1445 | 2472 | 1576 | 1813 | 1291 | 1770 | 2925 |
|  |  | 23% | 58% | 69% | 37% | 43% | 31% | 42% | 69% |
| Overall | number of studies  (%) | 18 | 45 | 57 | 29 | 33 | 19 | 21 | 63 |
|  |  | 17% | 58% | 69% | 35% | 40% | 23% | 25% | 76% |
|  | number of participants (%) | 1382 | 3437 | 5531 | 2285 | 2624 | 1683 | 2395 | 5345 |
|  |  | 17% | 43% | 68% | 27% | 32% | 20% | 29% | 64% |
