# Supplementary material for: Survival from alcoholic hepatitis has not improved over time
Source: PLoS One. 2018 Feb 14;13(2):e0192393. doi: 10.1371/journal.pone.0192393 (PMC5812634; doi:10.1371/journal.pone.0192393)
Supplement: S1 References — (DOCX) [file pone.0192393.s010.docx]

**Supplementary Information references**

1. Helman RA, Temko MH, Nye SW et al. Alcoholic Hepatitis. Natural History and Evaluation of Prednisolone Therapy. Ann Intern Med. 1971;74(3):311-321.

2. Porter HP, Simon FR, Pope CE et al. Corticosteroid Therapy in Severe Alcoholic Hepatitis. A Double-Blind Drug Trial. N Engl J Med. 1971;284(24):1350-1355.

3. Campra JL, Hamlin EM, Kirshbaum RJ et al. Prednisone Therapy of Acute Alcoholic Hepatitis. Report of a Controlled Trial. Ann Intern Med. 1973;79(5):625-631.

4. Blitzer BL, Mutchnick MG, Joshi PH et al. Adrenocorticosteroid Therapy in Alcoholic Hepatitis. A Prospective, Double-Blind Randomized Study. Am J Dig Dis. 1977;22(6):477-484.

5. Lesesne HR, Bozymski EM, Fallon HJ. Treatment of Alcoholic Hepatitis With Encephalopathy. Comparison of Prednisolone With Caloric Supplements. Gastroenterology. 1978;74(2 Pt 1):169-173.

6. Maddrey WC, Boitnott JK, Bedine MS et al. Corticosteroid Therapy of Alcoholic Hepatitis. Gastroenterology. 1978;75(2):193.

7. Shumaker JB, Resnick RH, Galambos JT et al. A Controlled Trial of 6-Methylprednisolone in Acute Alcoholic Hepatitis. With a Note on Published Results in Encephalopathic Patients. Am J Gastroenterol. 1978;69(4):443-449.

8. Depew W, Boyer T, Omata M et al. Double-Blind Controlled Trial of Prednisolone Therapy in Patients With Severe Acute Alcoholic Hepatitis and Spontaneous Encephalopathy. Gastroenterology. 1980;78(3):524-529.

9. Nasrallah SM, Galambos JT. Aminoacid Therapy of Alcoholic Hepatitis. Lancet. 1980;2(8207):1276-1277.

10. Baker AL, Jaspan JB, Haines NW et al. The University of Chicago Medical House-Staff. A Randomized Clinical Trial of Insulin and Glucagon Infusion for Treatment of Alcoholic Hepatitis: Progress Report in 50 Patients. Gastroenterology. 1981;80:1410-1414.

11. Halle P, Pare P, Kaptein E et al. Double-Blind, Controlled Trial of Propylthiouracil in Patients With Severe Acute Alcoholic Hepatitis. Gastroenterology. 1982;82(5 Pt 1):925.

12. Theodossi A, Eddleston AL, Williams R. Controlled Trial of Methylprednisolone Therapy in Severe Acute Alcoholic Hepatitis. Gut. 1982;23(1):75.

13. Mendenhall CL, Anderson S, Garcia-Pont P et al. Short-Term and Long-Term Survival in Patients With Alcoholic Hepatitis Treated With Oxandrolone and Prednisolone. New England Journal of Medicine. 1984;311(23):1464-1470.

14. Calvey H, Davis M, Williams R. Controlled Trial of Nutritional Supplementation, With and Without Branched Chain Amino Acid Enrichment, in Treatment of Acute Alcoholic Hepatitis. J Hepatol. 1985;1(2):141-151.

15. Achord JL. A Prospective Randomized Clinical Trial of Peripheral Amino Acid--glucose Supplementation in Acute Alcoholic Hepatitis. American Journal of Gastroenterology. 1987

16. Fehér J, Cornides Á, Romány A et al. A Prospective Multicenter Study of Insulin and Glucagon Infusion Therapy in Acute Alcoholic Hepatitis. Journal of hepatology. 1987;5(2):224-231.

17. Simon D, Galambos JT. A Randomized Controlled Study of Peripheral Parenteral Nutrition in Moderate and Severe Alcoholic Hepatitis. J Hepatol. 1988;7(2):200-207.

18. Carithers Jr RL, Herlong HF, Diehl AM et al. Methylprednisolone Therapy in Patients With Severe Alcoholic Hepatitis. A Randomized Multicenter Trial. Annals of internal medicine. 1989;110(9):685.

19. Akriviadis EA, Steindel H, Pinto PC et al. Failure of Colchicine to Improve Short-Term Survival in Patients With Alcoholic Hepatitis. Gastroenterology. 1990;99(3):811-818.

20. Mezey E, Caballería J, Mitchell MC et al. Effect of Parenteral Amino Acid Supplementation on Short-Term and Long-Term Outcomes in Severe Alcoholic Hepatitis: A Randomized Controlled Trial. Hepatology. 1991;14(6):1090-1096.

21. Bird G, Lau JY, Koskinas J et al. Insulin and Glucagon Infusion in Acute Alcoholic Hepatitis: A Prospective Randomized Controlled Trial. Hepatology. 1991;14(6):1097-1101.

22. Trinchet JC, Balkau B, Poupon RE et al. Treatment of Severe Alcoholic Hepatitis By Infusion of Insulin and Glucagon: A Multicenter Sequential Trial. Hepatology. 1992;15(1):76-81.

23. Ramond MJ, Poynard T, Rueff B et al. A Randomized Trial of Prednisolone in Patients With Severe Alcoholic Hepatitis. N Engl J Med. 1992;326(8):507-512.

24. Mendenhall CL, Moritz TE, Roselle GA et al. A Study of Oral Nutritional Support With Oxandrolone in Malnourished Patients With Alcoholic Hepatitis: Results of a Department of Veterans Affairs Cooperative Study. Hepatology. 1993;17(4):564-576.

25. Bird GL, Prach AT, Mcmahon AD et al. Randomised Controlled Double-Blind Trial of the Calcium Channel Antagonist Amlodipine in the Treatment of Acute Alcoholic Hepatitis. J Hepatol. 1998;28(2):194-198.

26. Cabre E, Rodriguez-Iglesias P, Caballeria J et al. Short- and Long-Term Outcome of Severe Alcohol-Induced Hepatitis Treated With Steroids Or Enteral Nutrition: A Multicenter Randomized Trial. Hepatology. 2000;32(1):36-42.

27. Akriviadis E, Botla R, Briggs W et al. Pentoxifylline Improves Short-Term Survival in Severe Acute Alcoholic Hepatitis: A Double-Blind, Placebo-Controlled Trial. Gastroenterology. 2000;119(6):1637-1648.

28. Spahr L, Rubbia-Brandt L, Frossard JL et al. Combination of Steroids With Infliximab Or Placebo in Severe Alcoholic Hepatitis: A Randomized Controlled Pilot Study. J Hepatol. 2002;37(4):448-455.

29. Mezey E, Potter JJ, Rennie-Tankersley L et al. A Randomized Placebo Controlled Trial of Vitamin E for Alcoholic Hepatitis. Journal of hepatology. 2004;40(1):40-46.

30. Naveau S, Chollet‐martin S, Dharancy S et al. A Double‐blind Randomized Controlled Trial of Infliximab Associated With Prednisolone in Acute Alcoholic Hepatitis. Hepatology. 2004;39(5):1390-1397.

31. Phillips M, Curtis H, Portmann B et al. Antioxidants Versus Corticosteroids in the Treatment of Severe Alcoholic Hepatitis--a Randomised Clinical Trial. Journal of hepatology. 2006;44(4):784-790.

32. Stewart S, Prince M, Bassendine M et al. A Randomized Trial of Antioxidant Therapy Alone Or With Corticosteroids in Acute Alcoholic Hepatitis. J Hepatol. 2007;47(2):277-283.

33. Boetticher NC, Peine CJ, Kwo P et al. A Randomized, Double-Blinded, Placebo-Controlled Multi-Center Trial of Etanercept in the Treatment of Alcoholic Hepatitis. Gastroenterology. 2008;135(6):1953.

34. De BK, Gangopadhyay S, Dutta D et al. Pentoxifylline Versus Prednisolone for Severe Alcoholic Hepatitis: A Randomized Controlled Trial. World Journal of Gastroenterology. 2009;15(13):1613.

35. Moreno C, Langlet P, Hittelet A et al. Enteral Nutrition With Or Without N-Acetylcysteine in the Treatment of Severe Acute Alcoholic Hepatitis: A Randomized Multicenter Controlled Trial. Journal of hepatology. 2010;53:1117-1122.

36. Nguyen-Khac E, Thierry Thevenot, M-AP et al. Glucocorticoids Plus N-Acetylcysteine in Severe Alcoholic Hepatitis. New England Journal of Medicine. 2011;365:1781-1789.

37. Sidhu SS, Goyal O, Singla P et al. Corticosteroid Plus Pentoxifylline is Not Better Than Corticosteroid Alone for Improving Survival in Severe Alcoholic Hepatitis (Cope Trial). Dig Dis Sci. 2012;57(6):1664-1671.

38. Sidhu SS, Goyal O, Singla M et al. Pentoxifylline in Severe Alcoholic Hepatitis: A Prospective, Randomised Trial. J Assoc Physicians India. 2012;60:20-22.

39. Singh V, Sharma AK, Narasimhan RL et al. Granulocyte Colony-Stimulating Factor in Severe Alcoholic Hepatitis: A Randomized Pilot Study. Am J Gastroenterol. 2014;109(9):1417-1423.

40. Higuera-De La Tijera F, Servín-Caamaño AI, Cruz-Herrera J et al. Treatment With Metadoxine and Its Impact on Early Mortality in Patients With Severe Alcoholic Hepatitis. Ann Hepatol. 2014;13(3):343-352.

41. Park SH, Kim DJ, Kim YS et al. Pentoxifylline Vs. Corticosteroid to Treat Severe Alcoholic Hepatitis: A Randomised, Non-Inferiority, Open Trial. J Hepatol. 2014;61(4):792-798.

42. Thursz MR, Richardson P, Allison M et al. Prednisolone Or Pentoxifylline for Alcoholic Hepatitis. N Engl J Med. 2015;372(17):1619-1628.

43. Moreno C, Deltenre P, Senterre C et al. Intensive Enteral Nutrition is Ineffective for Individuals With Severe Alcoholic Hepatitis Treated With Corticosteroids. Gastroenterology. 2016

44. Tkachenko P, Maevskaya M, Pavlov A et al. Prednisolone Plus S-Adenosil-l-methionine in Severe Alcoholic Hepatitis. Hepatology …. 2016

45. Chedid A, Mendenhall, Cl, Tosch T et al. Significance of Megamitochondria in Alcoholic Liver Disease. Gastroenterology. 1986;90:1858-1864.

46. Sheron N, Bird G, Goka J et al. Elevated Plasma Interleukin-6 and Increased Severity and Mortality in Alcoholic Hepatitis. Clinical and experimental immunology. 1991;84(3):449.

47. Hill DLB, Marsano LS, Mcclain CJ. Increased Plasma Interleukin 8 Concentrations in Alcoholic Hepatitis. Hepatology. 1993;18(3):576-580.

48. Fang JWS, Davis GL, Lau JYN et al. Hepatocyte Proliferation as an Indicator of Outcome in Acute Alcoholic Hepatitis. The Lancet. 1994;343(8901):820-823.

49. Rodriguez-Rodriguez E, González-Reimers E, Santolaria-Fernandez F et al. Cytokine Levels in Acute Alcoholic Hepatitis: A Sequential Study. Drug and alcohol dependence. 1995;39(1):23-27.

50. Sheth M, Riggs M, Patel T. Utility of the Mayo End-Stage Liver Disease (Meld) Score in Assessing Prognosis of Patients With Alcoholic Hepatitis. BMC gastroenterology. 2002;2(1):2.

51. Spahr L, Giostra E, Frossard J-L et al. Soluble Tnf-R1, But Not Tumor Necrosis Factor Alpha, Predicts the 3-Month Mortality in Patients With Alcoholic Hepatitis. Journal of hepatology. 2004;41(2):229-234.

52. Cuthbert JA, Arslanlar S, Yepuri J et al. Predicting Short-Term Mortality and Long-Term Survival for Hospitalized Us Patients With Alcoholic Hepatitis. Dig Dis Sci. 2014;59(7):1594-1602.

53. Dunn W, Jamil LH, Brown LS et al. Meld Accurately Predicts Mortality in Patients With Alcoholic Hepatitis. Hepatology. 2005;41(2):353-358.

54. Forrest EH, Evans CD, Stewart S et al. Analysis of Factors Predictive of Mortality in Alcoholic Hepatitis and Derivation and Validation of the Glasgow Alcoholic Hepatitis Score. Gut. 2005;54(8):1174-1179.

55. Louvet A, Naveau S, Abdelnour M et al. The Lille Model: A New Tool for Therapeutic Strategy in Patients With Severe Alcoholic Hepatitis Treated With Steroids. Hepatology. 2007;45(6):1348-1354.

56. Dominguez M, Rincon D, Abraldes JG et al. A New Scoring System for Prognostic Stratification of Patients With Alcoholic Hepatitis. Am J Gastroenterol. 2008;103(11):2747-2756.

57. Di Mambro AJ, Parker R, Mccune A et al. In Vitro Steroid Resistance Correlates With Outcome in Severe Alcoholic Hepatitis. Hepatology. 2011;53:1316-1322.

58. Sandahl TD, Jepsen P, Ott P et al. Validation of Prognostic Scores for Clinical Use in Patients With Alcoholic Hepatitis. Scand J Gastroenterol. 2011;46(9):1127-1132.

59. Spahr L, Rubbia-Brandt L, Genevay M et al. Early Liver Biopsy, Intraparenchymal Cholestasis, and Prognosis in Patients With Alcoholic Steatohepatitis. BMC Gastroenterol. 2011;11:115.

60. Pang JX, Ross E, Borman MA et al. Risk Factors for Mortality in Patients With Alcoholic Hepatitis and Assessment of Prognostic Models: A Population-Based Study. Can J Gastroenterol Hepatol. 2015;29(3):131-138.

61. Sancho Bru P, Altamirano J, Rodrigo Torres D et al. Liver Progenitor Cell Markers Correlate With Liver Damage and Predict Short Term Mortality in Patients With Alcoholic Hepatitis. Hepatology. 2012;55(6):1931-1941.

62. Lafferty H, Stanley AJ, Forrest EH. The Management of Alcoholic Hepatitis: A Prospective Comparison of Scoring Systems. Alimentary Pharmacology & Therapeutics. 2013;38(6):603-610.

63. Potts JR, Goubet S, Heneghan MA et al. Determinants of Long-Term Outcome in Severe Alcoholic Hepatitis. Aliment Pharmacol Ther. 2013;38(6):584-595.

64. Monsanto P, Almeida N, Lrias C et al. Evaluation of Meld Score and Maddrey Discriminant Function for Mortality Prediction in Patients With Alcoholic Hepatitis. Hepato-gastroenterology. 2013;(60):1089-1094.

65. Altamirano J, Miquel R, Katoonizadeh A et al. A Histologic Scoring System for Prognosis of Patients With Alcoholic Hepatitis. Gastroenterology. 2014;146(5):1231-1239. e6.

66. Papastergiou V, Tsochatzis EA, Pieri G et al. Nine Scoring Models for Short Term Mortality in Alcoholic Hepatitis: Cross Validation in a Biopsy Proven Cohort. Alimentary pharmacology & therapeutics. 2014;39(7):721-732.

67. Goyal SK, Dixit VK, Jain AK et al. Assessment of the Model for End-Stage Liver Disease (Meld) Score in Predicting Prognosis of Patients With Alcoholic Hepatitis. J Clin Exp Hepatol. 2014;4(1):19-24.

68. Kadian M, Kakkar R, Dhar M et al. Model for End Stage Liver Disease Score Versus Maddrey Discriminant Function Score in Assessing Short Term Outcome in Alcoholic Hepatitis. Journal of gastroenterology and hepatology. 2014;29(3):581-588.

69. Mazzocco T, Hussain A, Hussain S et al. A Novel Mortality Model for Acute Alcoholic Hepatitis Including Variables Recorded After Admission to Hospital. Computers in biology and medicine. 2014;44:132-135.

70. Rachakonda V, Gabbert C, Raina A et al. Stratification of Risk of Death in Severe Acute Alcoholic Hepatitis Using a Panel of Adipokines and Cytokines. Alcohol Clin Exp Res. 2014;38(11):2712-2721.

71. Lee M, Kim W, Choi Y et al. Spontaneous Evolution in Bilirubin Levels Predicts Liver-Related Mortality in Patients With Alcoholic Hepatitis. PloS one. 2014;9(7):e100870.

72. Michelena J, Altamirano J, Abraldes JG et al. Systemic Inflammatory Response and Serum Lipopolysaccharide Levels Predict Multiple Organ Failure and Death in Alcoholic Hepatitis. Hepatology. 2015

73. Gustot T, Maillart E, Bocci M et al. Invasive Aspergillosis in Patients With Severe Alcoholic Hepatitis. Journal of hepatology. 2014;60(2):267-274.

74. Sersté T, Njimi H, Degré D et al. The Use of Beta-Blockers is Associated With the Occurrence of Acute Kidney Injury in Severe Alcoholic Hepatitis. Liver Int. 2015;35(8):1974-1982.

75. Andrade P, Silva M, Rodrigues S et al. Alcoholic Hepatitis Histological Score Has High Accuracy to Predict 90-Day Mortality and Response to Steroids. Dig Liver Dis. 2016;48(6):656-660.

76. Ravi S, Bade KS, Hasanin M et al. Ammonia Level At Admission Predicts in-Hospital Mortality for Patients With Alcoholic Hepatitis. Gastroenterol Rep (Oxf). 2016

77. Beisel C, Blessin U, Schulze Zur Wiesch J et al. Infections Complicating Severe Alcoholic Hepatitis: Enterococcus Species Represent the Most Frequently Identified Pathogen. Scand J Gastroenterol. 20161-7.
